# Peripheral NK cell phenotypic alteration and dysfunctional state post hepatitis B subviral particles stimulation in CHB patients: evading immune surveillance

**DOI:** 10.3389/fimmu.2024.1427519

**Published:** 2024-09-12

**Authors:** Mohamed A Selim, Reda A. Suef, Ebrahim Saied, Mostafa A. Abdel-Maksoud, Saeedah Musaed Almutairi, Mohammed Aufy, Adel A. Mousa, Mohamed T. M. Mansour, Mohamed M. S. Farag

**Affiliations:** ^1^ Botany and Microbiology Department, Faculty of Science, Al-Azhar University, Cairo, Egypt; ^2^ Department of Botany and Microbiology, College of Science, King Saud University, Riyadh, Saudi Arabia; ^3^ Department of Pharmaceutical Sciences, Division of Pharmacology and Toxicology, University of Vienna, Vienna, Austria; ^4^ Virology and Immunology Department, National Cancer Institute, Cairo University and Childern’s Cancer Hospital, Cairo, Egypt; ^5^ Biomedical Research Department, Armed Forces College of Medicine (AFCM), Cairo, Egypt; ^6^ The Regional Centre for Mycology and Biotechnology, Al-Azhar University, Cairo, Egypt

**Keywords:** chronic hepatitis B (CHB), HB subviral particles (HBVsvp), HbsAg, natural killer (NK) cells, NKp46 and NKG2D activating receptors, CD94 inhibitory receptor, cytokine, immunotherapy

## Abstract

**Background:**

The relationship between chronic hepatitis B (CHB) infection and natural killer (NK) cell dysfunction is well-established, but the specific role of HBV viral antigens in driving NK cell impairment in patients with CHB remains unclear. This study investigates the modulatory effects of hepatitis B virus subviral particles (HBVsvp, a representative model for HBsAg) on the phenotypic regulation (activating and inhibitory receptors), cytokine production and cytotoxic potential of peripheral blood mononuclear cell-derived natural killer cells (PBMCs-derived NK cell), which contributes to NK cell dysfunction in CHB infection, potentially serving as an effective HBV immune evasion strategy by the virus.

**Methods:**

NK cells were isolated from peripheral blood of patients with CHB (n=5) and healthy individuals (n=5), stimulated with HBVsvp. Subsequent flow cytometric characterization involved assessing changes in activating (NKp46 and NKG2D) and inhibitory (CD94) receptors expression, quantifying TNF-α and IFN- γ cytokine secretion, and evaluating the cytotoxic response against HepG2.2.15 cells with subsequent HBVsvp quantification.

**Results:**

In CHB patients, *in vitro* exposure of PBMCs-derived NK cell with HBVsvp (represent HBsAg model) significantly reduced NK cell-activating receptors expression (P = 0.022), increased expression of CD94 ^+^ NK cells (p = 0.029), accompanied with a reduced TNF-α - IFN-γ cytokine levels, and impaired cytotoxic capacity (evidenced by increased cell proliferation and elevated HBVsvp levels in co-cultures with HepG2.2.15 cells in a time-dependent), relative to healthy donors.

**Conclusion:**

These findings suggest that HBVsvp may induce dysfunctional NK cell responses characterized by phenotypic imbalance with subsequent reduction in cytokine and cytotoxic levels, indicating HBVsvp immunosuppressive effect that compromises antiviral defense in CHB patients. These data enhance our understanding of NK cell interactions with HBsAg and highlight the potential for targeting CD94 inhibitory receptors to restore NK cell function as an immunotherapeutic approach. Further clinical research is needed to validate these observations and establish their utility as reliable biomarkers.

## 1 Introduction

In 2016, the World Health Organization set a goal to eliminate viral hepatitis as a public health concern by 2030 (1). Chronic hepatitis B virus (CHBV) infection is a major global health burden with over 250 million individuals globally having CHBV infection, linked to an annual death rate of about one million patients worldwide (2). Due to factors like the host immune response, which is influenced by viral persistence and chronic inflammation, patients with CHB are at a high risk of developing hepatocellular carcinoma (HCC) and liver cirrhosis. These factors also have a remarkable effect on the outcome of HBV infection and the efficacy of antiviral treatment (3).

In a 1970 investigation by Sherlock et al., five HCC patients tested positive for HBV surface antigen (HBsAg), providing the first indication of a connection between chronic HBV infection and HCC.

Compared to people with cirrhosis or chronic hepatitis, the prevalence of HBsAg in HCC patients is significantly higher, suggesting that the virus has a particular carcinogenic effect (4).

Natural HBV infection is characterized by the production of an enormous excess of non-infectious HB subviral particles (HBVsvp), which are produced *in vivo* or *in vitro* and have a diameter of 22–25 nm. These particles only contain the outer envelope lipid bilayer of the virion, which represents HBsAg that forms the viral envelope. They are produced a 1,000–100,000 times over the complete infectious virions. These patient-derived SVPs are immunogenic and were utilized in the initial HBV vaccination (5).

In chronic HBV infection, the relationship between immunological tolerance and the HBVsvp has been underlined (6). Research has indicated that HBVsvp may impede the immune response and promote HBV infection in addition to potentially contributing to the immunological tolerance state (7). By attaching to host neutralizing antibodies, HBV can act as a decoy and allow infectious Dane particles to enter liver cells (8). It can also exert significant degrees of inhibition, which allows HBV to elude the host immune response and maintain persistent infection (9).

The complex immunological interactions determine the outcome of HBV infection (clearance or chronicity). Early innate immune responses are crucial for limiting viral replication and activating adaptive immunity (10). However, HBV can circumvent this natural defense, resulting in persistent infections (11). Thus, improving the early innate response may be a method for limiting viral dissemination and preventing chronic HBV infection.

Natural killer (NK) cells are regarded as critical effectors in the early stages of the innate immune response to viral infection. NK cells can exert cytotoxic effects on virus-infected or malignant cells via mechanisms such as antibody-independent pathways involving perforin and granzymes, as well as antibody-dependent cytotoxicity of cells. Additionally, they create antiviral cytokines and chemokines (12). Instead of using antigen-specific receptors, NK cells use the “missing self” approach to detect targets and react to cells that lack typical markers (13). The harmonization of both activating and inhibitory signals governs NK cell responses (14). Key receptors, such as NKp46 (15) and NKG2D (16), activate NK cells and direct them to attack abnormal cells. NKG2D responds particularly to stressed or transformed cells with a reduced MHC class I molecules (17). The KIR/CD158 inhibitory receptor family, conversely, inhibits NK cells from attacking normal cells, hence maintaining immune system balance (18).

Upon recognition to the “stressed” cells, such as viral infected or tumor cells. NK cells can display cytolytic activity by releasing of lytic granules, or by triggering apoptosis of infected cells (19). In addition to cytolytic potential, NK cells also play a significant role in the early stages of the innate immune response by producing interferon (IFN)-γ, interleukin (IL)-10, tumor necrosis factor (TNF)-α, granulocyte macrophage colony-stimulating factor (GM-CSF), and granulocyte colony-stimulating factor (G-CSF) (20).

Acute HBV infection causes NK cells to become activated, which in turn triggers adaptive immunity, exhibits antiviral properties, and may influence HBV specific CD8+ T cells. Converly in chronic HBV infection, NK cells display a complicated spectrum of functional selective deficiencies, including intact cytolytic activity but defect cytokine production, which may aid in the virus’s persistence (21–23). Also, prolonged HBV infection modifies NK cell receptors expression, upregulates inhibitory receptors like NKG2A, and downregulates activating receptors such as CD16 and NKp30, changes that correlate with the viral load. This receptors modification is subject to debate, with conflicting reports on NKG2A levels during chronic infections (24).

Currently, two antiviral drugs are available for treating CHB infection: nucleus(t)ide analogs (NUCs) and interferons (IFNs). However, these therapies require long-term medication and seldom achieve functional cures for chronic infectionsusually fail to clear HBsAg in these patients (25). Therefore, developing new therapeutics with improved safety and efficacy is necessary. NK cell-based immunotherapy, including NK cell therapy, checkpoint inhibition therapy, and others, has proved safe and effective against cancers (26, 27). Immune-checkpoint inhibitors, whose therapeutic value has been revealed in the field of cancer therapy, could potentially relieve suppression of NK cells’ activity in (CHB) by preventing inhibitory signaling through inhibitory checkpoint receptors (28, 29).

While the impact of HBV on NK cells in people with CHB is well documented, there is limited understanding of how antigens encoded with HBV, like HBsAg, affect NK cell activity. Research has indicated a decrease in hepatic NK cells and weakened cytotoxicity in mouse chronic carriers of HbsAg (30). Furthermore, the hepatic NK cells’ reaction to certain stimulations, such as poly (I:C), is significantly compromised within these carriers, leading to reduced antitumor activity and impaired reaction to viral stimulation (10). Additionally, in individuals immunized with HBsAg, NK cells are consistently activated and produce increased levels of IFN-γ. Conversely, in non-responders, NK cell activity was reduced, along with diminished production of regulatory cytokines (21). This emphasizes the role of HBsAg not only as a biomarker for HBV replication, but also as a contributor to immunopathogenesis during persistent HBV infection. Thus, the goal of the current study was to look into *vitro* interaction of HBV-encoded HbsAg (represented by HBVsvp in our study) with NK cell-activating receptors (NKp46; NKG2D), inhibitory receptors (CD94) in the peripheral blood of both healthy and CHB patients individuals, and whether this interaction influenced NK cell function and response using flow cytometry, measuring cytokine production (TNF-α - IFN-γ) in the supernatant. In addition, we assessed the cytotoxic role of HBVsvp-pulsed NK cells against virus-infected cells (HepG2.2.15 cell line). By understanding this interaction, we hope to improve NK cell performance, and could be a potential NK-based immunotherapeutic strategy for CHB patients.

## 2 Materials and methods

### 2.1 NK cells isolation approaches

#### 2.1.1 Cell lines and reagents

Dr. Stefan Urban supplied HepG2.2.15. (Heidelberg University, Germany) and maintained in Williams’ E medium (Invitrogen, USA), which has been enhanced with 10% (v/v) FCS (Invitrogen, USA), 2 mL glutamine, 100 U of penicillin/mL, 100 μg of streptomycin/mL, 5 μg/mL insulin, and 5 μg/mL hydrocortisone (Sigma-Aldrich, USA). To determine the HBVsvp concentration, the polyethylene glycol protocol was applied. HBVsvp were tested using ELISA for HBsAg positive (Cam Tech Medical, UK), For peripheral blood mononuclear cells (PBMCs) separation, we used the Ficoll-plaque centrifugation using a density gradient technique. To summarize, 1x Balanced Salt Solution was used to dilute the blood samples (Gibco, UK) at a 1:3 ratio, diluted cell suspension layered over Ficoll- Hypaque (GE Healthcare, Life Science, Sweden)

NK cell analysis was carried out using a multicolor FACS Calibur (BD Biosciences, USA). The NK cell population was identified using CD56PE (, BD Biosciences, USA - Cat. no. 561903, lot no. 8141891) and CD3-perCP conjugation. The activation marker NKG2DAPC (CD314) (BD Biosciences, USA- Cat. no. 562064, lot no. 8142790), and inhibition marker CD94FITC (BD Biosciences, USA- Cat. no. 555889); were assessed in conjunction with CD56PE/CD3PerCP (BD Biosciences, USA- Cat. no. 95131, lot no. 345766) and NKp46 APC (CD335) (BD Biosciences, USA, Cat. no. 558051, lot no. 8151558).

For cytokine quantification, the study employed an AssayMax™ Human Interferon-gamma (IFN-gamma) Sandwich ELISA Kit (Assaypro, USA- Catalog No. EI1023-1) for IFN-γ and an AssayMax™ Human TNFα Sandwich ELISA Kit (Assaypro, USA - Catalog No. ET2010-1) for TNF-α. Assays were conducted in strictly in line with the guidelines provided by the manufacturer.

#### 2.1.2 Patient sample collection

The study was conducted at Egypt’s Children’s Cancer Hospital (57357) and Al-Zahra University Hospital’s clinical pathology laboratories. Ten human whole blood samples were collected (15 ml per sample) from both CHB patients (n = 5) and healthy donors (n=5) in a heparin-containing vacutainer as an anticoagulant (Greiner Bio-one, Australia). For CHB patients, the diagnosis was defined by a high level of plasma HBV DNA load, which was confirmed through real-time PCR. Biochemical analysis was performed using routine methods. All CHB patients were serologically tested using ELISA for the presence of serum envelope antigen (HBeAg) and surface antigen (HBsAg) of HBV, both of which were positive. All control subjects were negative for HIV, HBV, and HCV and were free of any conditions affecting the liver. The clinical features of CHB patients (with active viral replication) and healthy controls included in the study are outlined in **Table 1**. The CHB patients and control were age- and sex-matched. CHB patients who had undergone antiviral therapies (nucleos(t)ide analog or pegylated IFN-α) during the previous 6 months, those who were coinfected with HIV, HCV or HDV, drug/alcoholic-induced hepatitis, or who had immunological liver disorders, were excluded from the study. Informed consent was obtained, and the local ethics committee of the CCHE and the Liver Institute approved the recruitment of patients with CHB. Blood samples were obtained from the Al-Zahraa University Hospital. Isolation of NK cells was performed at the Tissue Culture Laboratory, Egypt’s Children’s Cancer Hospital (57357). Phenotypic identification of NK cells was performed at the Hematology Unit, Flow Cytometry Lab, Clinical Pathology Department, Al-Azhar University.

**Table 1:** Clinical features of the participants under study.

Clinical features	Chronic hepatitis B	Healthy donors
**Age, year [mean ± SEM]**	29 ± 2.1	28.6 ± 1.7
range	13	26
**Gender, n (%)**	(n = 5)	(n = 5)
Male	3 (60%)	3 (60%)
Female	2 (40%)	2 (40%)
**ALT U/ml [mean ± SEM]**	42.4 ± 4.6	26 ± 3.4
(range)	26	31
**AST (IU/mL) [mean ± SEM]**	42.4 ± 4.6	26 ± 3.4
(range)	19	9
**HBV load (IU/mL)**	1328.924	N.A.
Median	675.12	N.A.
**Total HBcAb (IgG)**	positive	N.A.
**HBeAg**	**positive**	N.A.
**HBsAg**	**positive**	N.A.
**Anti-HBs**	**Negative**	N.A.

SEM, standard error of mean; NA, not applicable; ALT, alanine aminotransferase; AST, aspartate transaminase; HBcAb, hepatitis B virus core antibody.

#### 2.1.3 HBVsvp harvesting

HBVsvp were harvested following our previous protocol (31). In brief, HepG2.2.15 cells were kept in Williams’ E medium (Invitrogen, USA). The HepG2.2.15 cells were then cultured at 37°C in a CO_2_ incubator for a definite period (for up to 7 days) resulting in significant production of HBVsvp in the supernatant, as seen in our past results (32).

The highest production rate of HBVsvp was determined for each over 7 days of HepG2.2.15 culturing separately, Briefly, cell culture supernatant (containing HBVsvp) at separate days (from1 to 7) were harvested for concentration by polyethylene glycol (PEG) protocol. Initially, all samples were incubated overnight at 4°C with polyethylene glycol PEG (40%) (Nice, India). On the second day, the medium was centrifuged at 4000 rpm for two hours at 4°C, the pellet was resuspended in a mix of 1x PBS (75%) and FCS (25%), followed by incubation for a second night at 4°C. On the third day, the supernatant (isolates) was harvested and centrifuged again at 4000 rpm for 20 minutes at 4°C. After centrifugation, the concentrated HBVsvp isolates were collected and stored at -20°C for quantification by ELISA. The highest HBVsvp production rate was determined quantitatively by ELISA, which revealed that, in comparison to concentrated HBVsvp on day one (1.1 O.D.), the maximum HBVsvp concentration was found in the day 7 concentrated sample (1.7 O.D.) Lastly, the supernatant containing HBVsvp was kept for future research at -80°C (33).

#### 2.1.4 Molecular characterization of HBVsvp

The RTP^®^ DNA/RNA Virus Mini Kit (Berlin, Germany) was used to extract the DNA of the HB virus following the manufacturer’s guidelines. The extracted DNA was stored at -80°C for downstream Nested PCR Assay for HBV X Gene amplification using two sets of forward and reverse primers. The amplification process was carried out using MyTaq Red Mix, 2x (Bioline, Luckenwalde, Germany). The tubes were then placed in an Applied Biosystem ProFlex™ thermocycler after optimizing the PCR cycling conditions following the manufacturer’s guidelines as follows: samples were first denatured at 95°C for 1 minute. This was followed by 35 cycles of denaturation at 95°C for 30 seconds, annealing at 56°C for 30 seconds, extending at 72°C for one minute, and extending again at 72°C for 10 minutes. Finally, samples were held at 4°C until the program was manually ended. Detection of the amplified HBV X gene PCR product was performed using 2μl of the second-round PCR product on agarose gel (2%) containing 10μl of ethidium bromide (10 mg/ml) using an electrophoresis chamber (Pharmacia, LKB). The expected HBV X gene band against the 100bp DNA ladder was 465 bp using UV transillumination.

### 2.2 NK cells isolation approach

#### 2.2.1 PBMCs-derived NK cell separation

PBMCs were obtained from both patients with CHB and whole blood from healthy donors using Ficoll gradient separation following centrifugation. To put it briefly, anticoagulated blood samples were diluted 1:3 in 1x Balanced Salt Solution (Gibco, UK), and 15 mL of Ficoll-Hypaque was placed over 35 mL of diluted cell suspension (blood/PBS mixture). (GE Healthcare, Life Science, Sweden). The Ficoll/diluted blood mixture was centrifuged at 1600 rpm (400xg) for 30 min at 20°C. After centrifugation, the four distinct layers represented the blood components that were separated according to their densities. For purification and harvesting of the PBMCs pellet (including NK cells), the PBMCs layer was collected, washed, resuspended, and centrifuged at different speeds for optimization. In addition, the PBMCs count per milliliter and viability (percentage of live cells) rate (seen as bright cells) were assessed using trypan blue exclusion vital staining. In this assay, 1:1 dilution was achieved by mixing an equal volume of 0.4% Trypan blue reagent and cell suspension. The mix was then transferred onto a hemocytometer chamber counting, with an average PBMCs cellular recovery of around 60% of the starting cell number, and the viability was around 90%.

#### 2.2.2 PBMCs-derived NK cell purification

For purification of NK cells from the patients with CHB and healthy donors, (1x10^6^ PBMC cells^/^ml) were suspended in 6 well plates in DMEM complete medium (Sigma-Aldrich, USA) and incubated for 2 h. Next, the non-adherent cells (including NK cells) were gently collected for phenotype and purity assessment using flow cytometry (BD Biosciences, USA) using CD3/CD56+CD16 fluorescent-conjugated monoclonal antibodies (34).

### 2.3 PBMCs-derived NK cells analysis

#### 2.3.1 Phenotype and frequency fluorometric assessment

Before starting the sorting technique, All samples were appropriately compensated using calibration beads (BD Biosciences, USA) to ensure accurate measurement of fluorescence intensity for each channel. Initially, PBMC-derived NK cells (2×10^5^) from CHB patients and healthy donors were purified by using BD Red blood cell lysis reagent for 5 min. To determine the frequency and phenotype of purified PBMCs-derived NK cells, multi-color flow cytometry was performed by staining the purified PBMCs-derived NK cells with 20µl of the following fluorochrome-labeled conjugated human antibodies cocktail: FITC-conjugated anti-CD3, PE-conjugated anti-CD16+CD56 (BD Biosciences, USA), for 25 min in dark incubation. Following a 3-minute centrifugation at 2000 rpm, the cells were twice cleaned using FACS buffer. (0.5% BSA-PBS). For sorting NK cells, first gating with forward and sideways scatters on a dot plot graph (FS/SS) was performed. Regarding the gating strategy of sorted PBMCs-derived NK isolate, gating was set to the lymphocytes’ predicted region (R1), and cells were analyzed using a quadrant plot, where the Y axis showed CD56+CD16 PE and the X axis showed CD3 FITC. Following gating on CD3 Negative cells and CD56+CD16 Positive cells, the sorted cells were collected later in sterile conical collection tubes (containing collection medium supplemented with FBS) [29]. The fluorescence baseline was determined using unstained cells. All analyses were carried out on a multi-color FACS Calibur flow cytometer (BD, Biosciences, USA). For data analysis, BD Biosciences’ Cell-Quest Pro software (San Jose, USA) was used. After isolation, NK cells were maintained in proper culturing conditions to ensure their viability and functionality until they were used for subsequent analysis.

#### 2.3.2 PBMCs-derived NK cells immunomodulation by HBVsvp pulsation

PBMC-derived NK cells immunomodulation induced by co-culturing with HBVsvp in a complete DMEM medium. Initially, we determined the optimal concentration of HBVsvp, used an PBMCs-derived NK immunostimulant (was at day 7 as previously mentioned), PBMCs-derived NK cells (1.5x10^6^ cells/ml) were co-cultured with a graduated concentration of HBVsvp (10,25,50 and 75µl). The plates were then incubated in the CO_2_ incubator for 12, 24, 48 and 72hr. The proliferation and viability of NK cells were evaluated using an inverted microscope each day. A 24-hour exposure time was found to be optimal, as it facilitated the maximization of NK cell activation while preserving cell viability. The prolonged exposure exceeding 24 hours led to a significant decline in cell viability, confirmed through morphological examination using an inverted microscope, which unveiled alterations suggestive of cellular stress. Following the incubation period, (1.5x10^5^ cells/ml) HBVsvp pulsed NK cells (derived from healthy individuals and CHB patients) from the 24hr exposure duration were propagated, washed, and stained to characterize the phenotype of NK cells following exposure to HBVsvp using flow cytometry. The supernatant obtained from each day’s culture was collected and stored at -20°C for subsequent measurement of cytokine production by NK cells.

#### 2.3.3 HBVsvp pulsed NK cells phenotypic profiling by fluorometric analysis

For phenotypic profiling of the activation, and inhibition, on HBVsvp pulsed NK cells using flow cytometry, the harvested HBVsvp pulsed NK cells from patients with CHB and healthy donors were initially titrated to determine the optimum fluorochrome concentrations. In brief, 50 ug of fresh sample after adjustment of cell count (1-5X10^6^/ml) was withdrawn in each of two stained tubes for 10 min at room temperature before final spin wash. 1st tube; CD56PE (BD Biosciences, USA Cat. no. 561903, lot no.8141891)/CD3PerCP (BD Biosciences, USA Cat. no. 95131, lot no.345766)/NKG2DAPC (CD314) (BD Biosciences, USA Cat. no. 562064, lot no.8142790); 2nd tube; CD94FITC (BD Biosciences, USA Cat. no. 555889)/CD56PE (BD Biosciences, USA Cat. no. 561903, lot no.8141891)/CD3PerCP (BD Biosciences, USA Cat. no. 95131, lot no.345766)/NKp46 APC(CD335) (BD Biosciences, USA Cat. no.558051, lot no.8151558); 3rd tube: non stained samples to detect auto-fluorescence. Fifty thousand events were acquired for analysis. Non-sub-viral particles treated cells were considered as controls. Single histograms were used to determine the percentage of positivity and mean fluorescence intensity of each marker expressed on NKs.

### 2.4 HBVsvp stimulated NK cells mediated functional evaluation

#### 2.4.1 Co-culture with HepG2.2.15 cells

To assess the functional significance of NK cells (from both healthy individuals and CHB patients) treated with varying doses of HBVsvp, we employed a human hepatoblastoma cell line HepG2.2.15 (transfected with HBV surface region as an *in vitro* expression system for HBVsvp representative to HBVsAg). Initially, both cell lines were maintained by culturing in 10 ml of complete Williams medium E (as previously mentioned) at 37°C in a CO_2_ incubator. After assessing 90% confluence for both cell lines, the HepG2.2.15 density and viability were determined using trypan blue assay. Subsequently, HepG2.2.15 cells were seeded into 96-well plates at a density of (5×10^3^ per well) and incubated for 24h at 37°C in a 5% CO_2_ incubator.

The following day, NK cells stimulated with HBVsvp (25×10^3^ cells/well) were loaded to pre-cultured HepG2.2.15 cells 96-well plates, while the control group of HepG2.2.15 cells lacked HBVsvp stimulated NK cells. Then, the co-cultured cells mixture was incubated in a 5% CO_2_ incubator at 37°C for durations of 24, 48, 72, and 96 hours. Each day, the cells were examined daily utilizing an inverted microscope to assess their morphological characteristics. Additionally, an absence of trypan blue dye test was employed to assess the vitality of cells. Subsequently, supernatant from each sample type and control group were collected to measure HBsAg and TNF- α and IFN-γ cytokine levels separately for each day using ELISA.

#### 2.4.2 Cytotoxicity assay

To evaluate the cytotoxicity mediated by HBVsvp-stimulated NK cells, HepG2.2.15 cells were employed as target cells at a target-to-stimulated (T: S) ratio of 1: 5 The MTT colorimetric assay was utilized in the following manner: 200 μL of MTT Reagent was added to each sample type and control group with different cell densities for each day separately. For two and a half hours, the plates were kept at 37°C in a humidified cell culture incubator. Using an inverted microscope, the cells were inspected for the appearance of intracellular dark purple needle-like formazan crystals. Once the purple precipitate was visible under a microscope, 200µl of detergent reagent (DMSO) was added to each well, and the formazan salt was allowed to dissolve in the dark for two to four hours at room temperature. Finally, using a reference wavelength of 650 nm, the absorbance of the solubilized formazan crystals was measured in each well using ELISA at a wavelength between 570 and 590 nm.

#### 2.4.3 Quantitative measuring HBsAg levels in HepG2.2.15 supernatant

After being cocultured for 24, 48, 72, and 96 hours, the HepG2.2.15 cell line’s response to coculturing with HBVsvp stimulated NK cells was evaluated by quantitatively measuring HBsAg levels in the collected supernatant using an ELISA assay following the instructions provided by the manufacturer (Cam Tech Medical; USA). The color development was then measured at a wavelength of 450 nm/reference wavelength of 630nm. The average values from duplicate readings were determined and adjusted by subtracting the average value for the cut-off.

#### 2.4.4 Cytokine measurement

Following stimulation of the NK cells with HBVsvp, we measured the levels of cytokines (TNF- α and IFN-γ) in the culture supernatants using ELISA kits (Assaypro, USA). According to the manufacture instructrion, All reagents, standards, and samples were prepared at room temperature (20-25°C). 50 µl of both human TNF- α and IFN-γ Standard/sample was added to each well, covered, and incubated for 2 hours. The microplate was washed manually (5x with 200 µl Wash buffer. 50 µl of Biotinylated Human TNF- α and IFN-γ Antibody was added to each well, covered, and incubated for 2 hours. The microplate was washed again. 50 µl of SP Conjugate was added to each well, covered, and incubated for 30 minutes. 50 µl of Chromogen Substrate was added, incubated for 25 minutes, then 50 µl of Stop Solution was added, and the absorbance was read at 450 nm. In the data analysis procedure, the mean value of the duplicate readings for each standard and sample was calculated. A standard curve was generated by plotting the standard concentrations on the x-axis and the corresponding mean 450 nm absorbance (OD) on the y-axis, with the best fit line determined. The unknown sample concentration was then determined from the standard curve. The concentrations of cytokines in pg/mL were determined based on the manufacturer’s provided standard reagents. The Kit demonstrates a high sensitivity and specificity levels, necessary for percise cytokine quantification. The kit’s sensitivity of 40 pg/ml for TNF- α and 0.25 pg/ml for IFN-γ. Also, it recognizes both natural and recombinant human TNF- α and IFN-γ specifically, underline its specificity. These performance characteristics underscore the reliability and accuracy of the assay in measuring TNF- α and IFN-γ levels in our samples.

### 2.5 Statistical analysis

The data were analyzed using IBM SPSS Statistics version 26. Descriptive statistics, including the mean and standard deviation, were calculated. Tow-way ANOVA was utilized with Tukey’s multiple comparisons test was used to compare groups for normally distributed quantitative variables. unpaired t-tests was utilised for comparing the means of two independent groups. P-values less than 0.05 were considered statistically significant In our study, the findings will be presented using various graphical formats to clearly illustrate the results. Dot plots will be used to identify and gate NK cell populations, histograms will display the expression levels of cell receptors, and scatter plots will represent the percentage and purity of NK cells within the PBMCs. Bar graphs will be utilized to depict the cytotoxic potential of NK cells, the dynamic interaction between HBVsvp-stimulated NK cells and HepG2.2.15 cells, the levels of HBVsvp in the supernatant, and the comparative analysis of IFN-γ and TNF-α cytokine secretion levels, with all the data presented as mean ± SEM, along with p-values to indicate the significance of the findings.

## 3 Results

### 3.1 Molecular characterization of HBVsvp (HBV-X gene detection)

**Figure 1:**
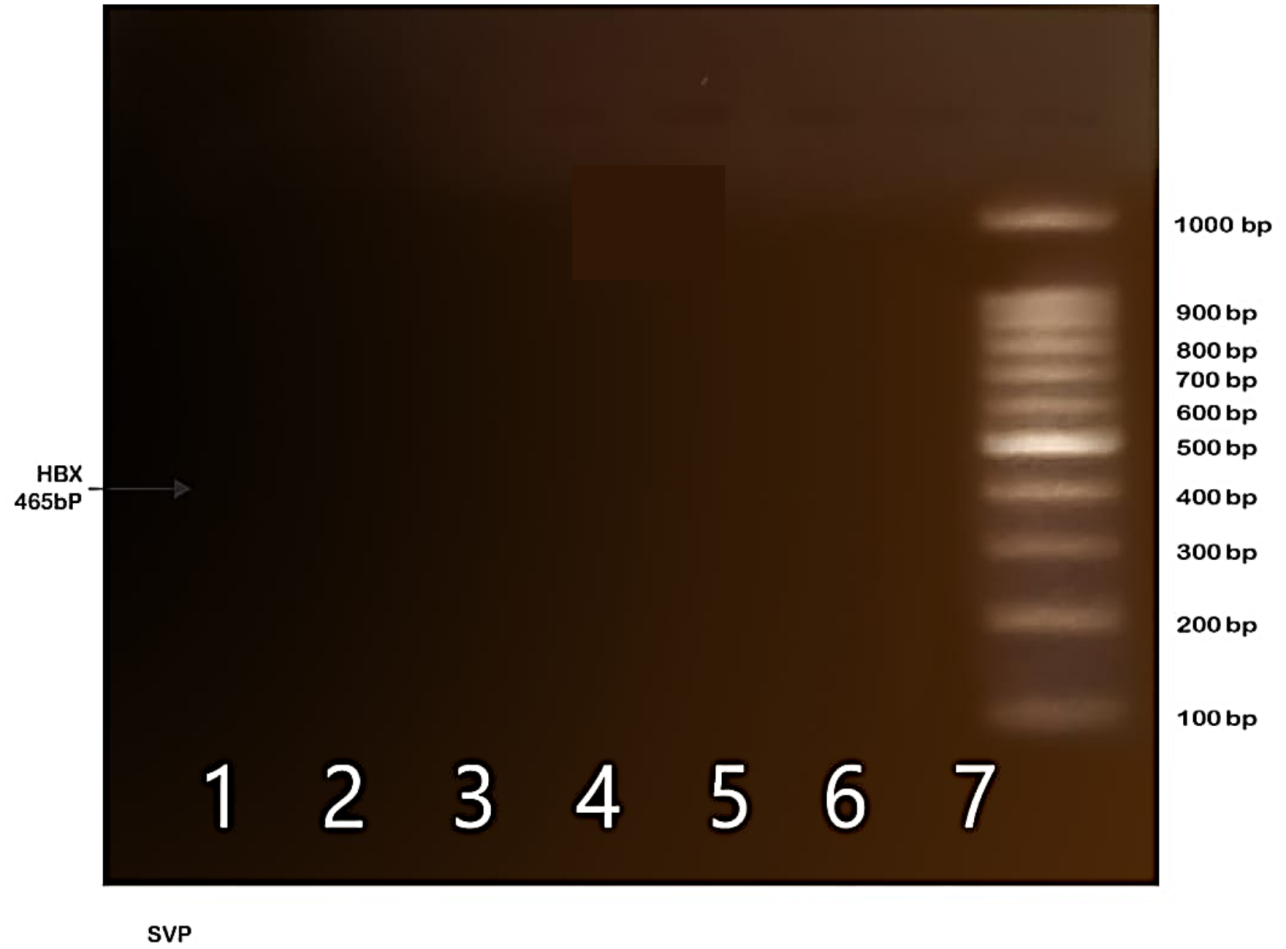
Indicates the absence of the HBX gene fragment (465 bp) in the SVP sample, suggesting the presence of empty SVP particles that serve as a model for HBsAg.

The Nested PCR characterization of HBVsvp- secreted by HepG2.2.15 cell line using a specific primer confirmed the presence of empty noninfectious HBVsvp (lacking the viral HBX gene at 465 bp fragment) compared to the complete HB virion (**Figure 1**).

### 3.2 PBMCs-derived NK cells analysis

#### 3.2.1 Identification of PBMCs-derived NK Population

**Figure 2:**
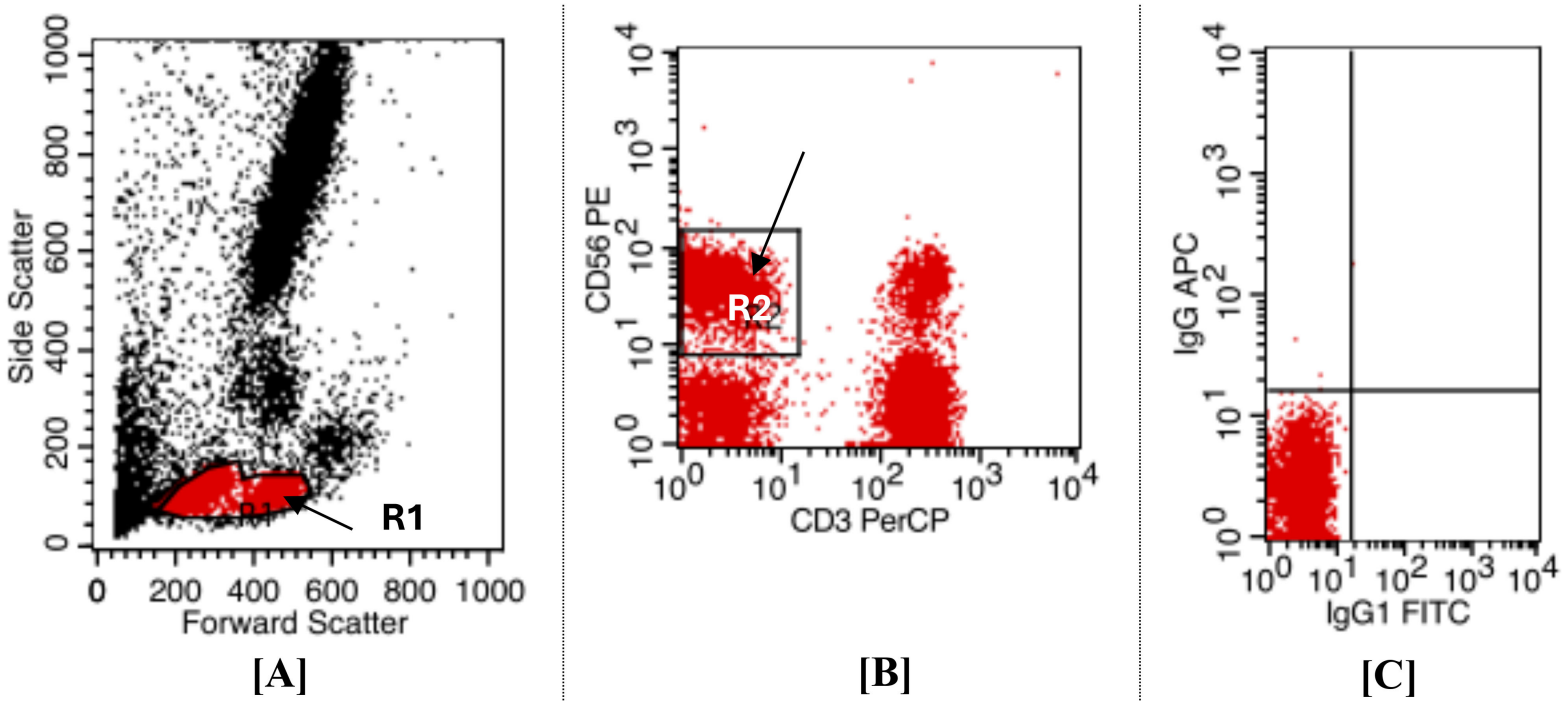
Displays flow cytometry dot plot analysis was employed to identify and gate NK cell populations from CHB patients and healthy donors, allowed for the gating zone to encompass both small and large lymphocyte-sized cells (R1) (gated area) distinct from other peripheral blood cells. This illustrates the gating method for NKs sorting; **(A)** Dot plot histogram for initial gating of lymphocyte region on FS/SS. **(B)** Identification of NKs. NKs were described as cells that were resident in (R2) and were (CD3 FITC negative/CD56+CD16 PE positive). **(C)** To set the cut-off positive for isotype control, use a quadrant plot. It was established that [R1 and R2] was the sorting gate.

For the definition of NK subsets based on surface marker expression, Using forward and sideway scatters (FS/SS) for the initial gating on the dot plot graph, the expected region of lymphocytes was the source of the gating (R1). (**Figure 2A**), After the cells were analyzed, a quadrant plot was created, with CD56+ PE found on the Y axis and CD3 PerCP on the X (**Figure 2B**). Based only on the expression of surface markers, NKs subsets were identified as CD56+CD16 positive and CD3 negative (**Figure 2C**). As per our plan, the NK population was identified as (R2) and located in the upper left (UL) quadrant of the map, indicated by a black arrow. The positivity cutoff was calculated using corresponding isotype control (sorting gate values were adjusted to R1 and R2 based on recovery mood). These findings align with our previous work, which established the initial gating strategy for NK cell sorting (34).

#### 3.2.2 NK frequency and purity fluorometric quantitative assessment

**Figure 3:**
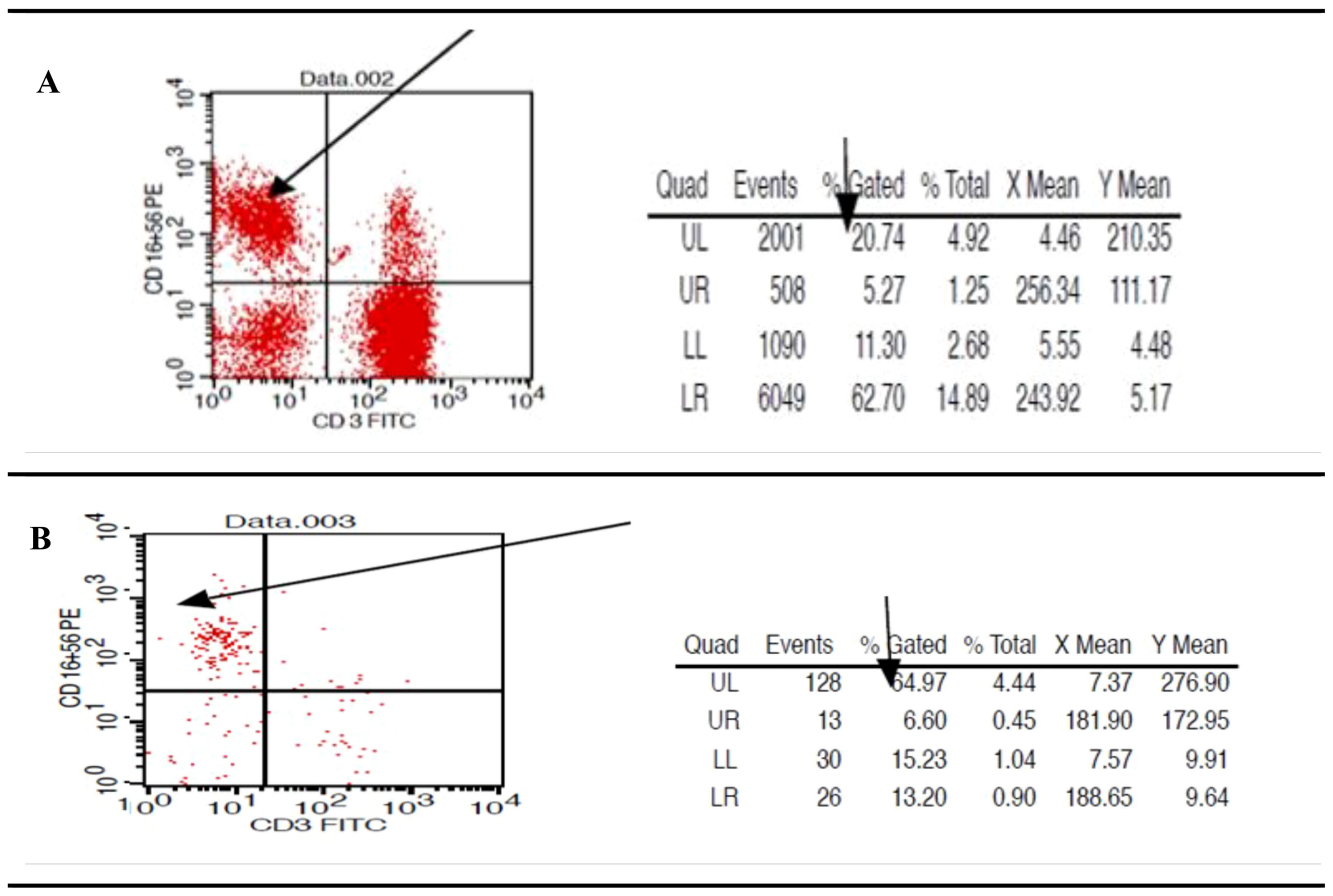
Presents flow cytometric scatter plot analysis to assess the percentage and purity of natural killer cells within the peripheral blood mononuclear cell fraction. **(A)** post-Ficoll density gradient separation, the abundance of NK cells, characterized by the CD56+CD16+ and CD3- phenotype, displaying variable levels across the evaluated samples. **(B)** Quantification of NK cell purity showed an average of 64.97% for the CD56+CD16+ and CD3- population in the selected samples.

Utilizing the Ficoll separation protocol, 10 milliliters of freshly drawn, heparinized blood were used to isolate peripheral blood mononuclear cells (PBMCs), which included a mixture of NK cells, other lymphocytes, and monocytes. These samples were then subjected to Phenotype fluorometric assessment to evaluate the purity post-centrifugation, following resuspension in 1 ml of PBS. The analysis revealed that NK cells constituted approximately 25% % of the PMBC mixture (**Figure 3A**) In terms of NK cell purity, the flow cytometry sorting technique was employed. The heparinized samples were stained and processed. This subsequent assessment demonstrated a consistent purity level of 64.97% % for NK cells that were positive for CD56 and CD16, and negative for CD3, across all samples examined as shown in the accompanying (**Figure 3B**). These findings align with our previous work, which established the initial gating strategy for NK cell sorting (34).

### 3.3 HBVsvp pulsed NK cells from CHB patients phenotypic profiling alteration

#### 3.3.1 NKG2D and NKp46 activating receptors

**Figure 4:**
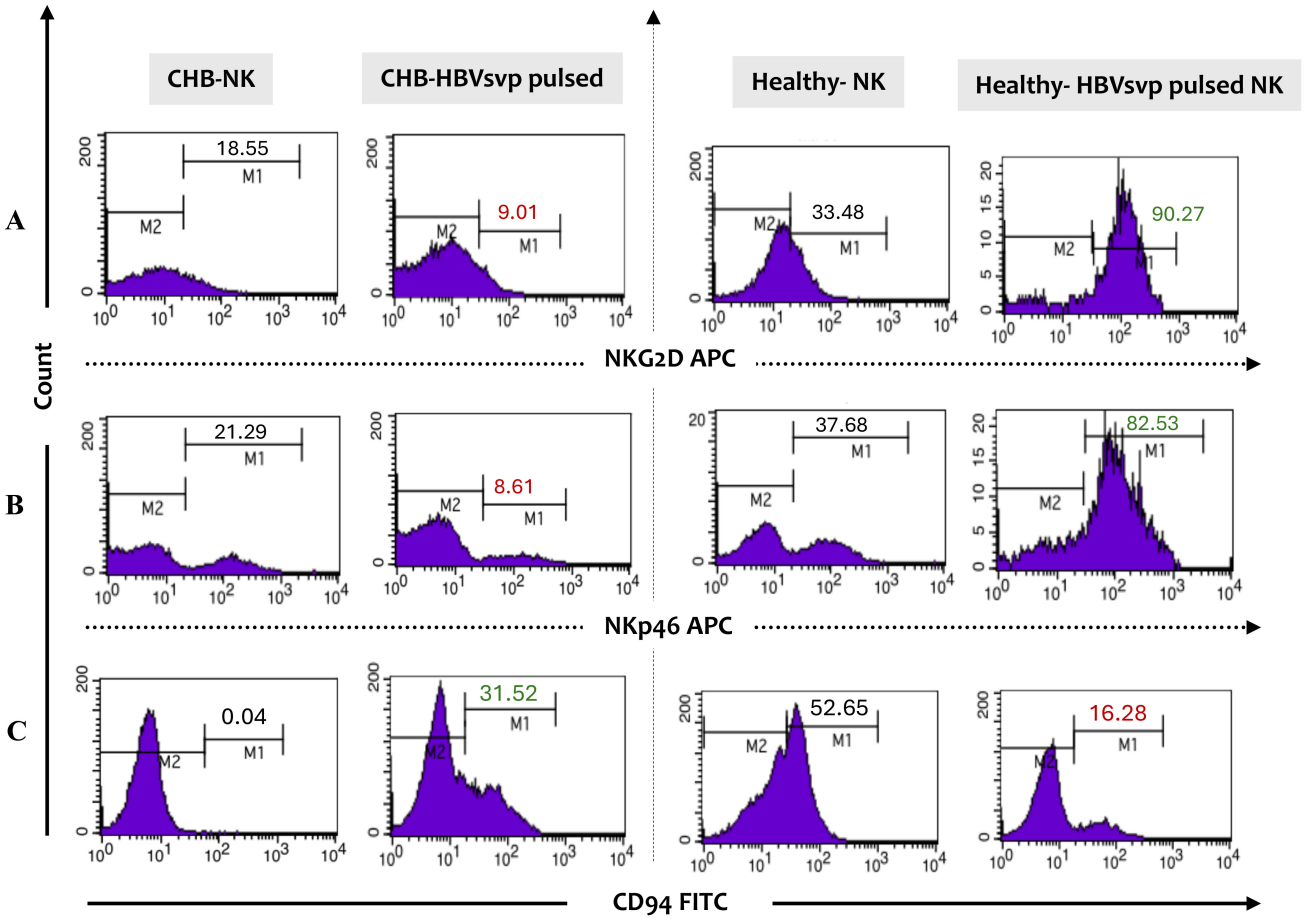
Presents a flow cytometry histogram analysis of the phenotypic expression profile of inhibitory and activating receptors on NK cells from CHB patients and healthy controls following stimulation with HBVsvp particles. The frequency analysis includes the expression levels of **(A)** the activating receptors NKG2D and **(B)** NKp46, as well as **(C)** the inhibitory receptor CD94 on gated NK cells. This provides insights into the functional state of NK cells in the context of HBV infection. The analysis was conducted using monoclonal antibodies directly conjugated with allophycocyanin (APC) and fluorescein isothiocyanate (FITC).

The diverse roles of NK cells, including their cytotoxic activity and the release of cytokine signaling proteins, are carefully controlled by the balance of both inhibitory and activating signals, which ultimately influence NK cell responses. To examine the phenotype profiling of NK cells, we assessed the expression levels of activating receptors (NKp46 and NKG2D) and inhibitory receptors (CD94) in NK cells from the peripheral blood of CHB patients and healthy individuals. Flow cytometry profiling results have shown significant differences in NK cell phenotype frequency between chronic HBV and healthy individuals. In patients with CHB, the activating receptors profiling frequency reported imbalanced receptor expression with a down-regulation NKG2D, NKp46 in HBVsvp pulsed NK cells relative to significantly increased percentage of NK expressing activation markers NKG2D, NKp46 (P Value = 0.022) derived healthy controls, these observed NK cells activation markers alterations frequently in CHB result in reduced NK cell capacity to eliminate HBV-infected cells, potentially impair HBV eradication (**Figures 4A, B**). Conversely, the phenotypic profiling findings revealed that CD94, an inhibitory marker, was significantly (p = 0.029) upregulated with an increased median value in HBVsvp pulsed NK cells obtained from chronic HBV patients compared to decreasing expression in NK cells derived from healthy donors, theses upregulation weaken NK cells killing response (**Figure 4C**).

#### 3.3.2 Impact of HBVsvp-stimulated NK cells on HepG2.2.15 cells viability- healthy donors

**Figure 5:**
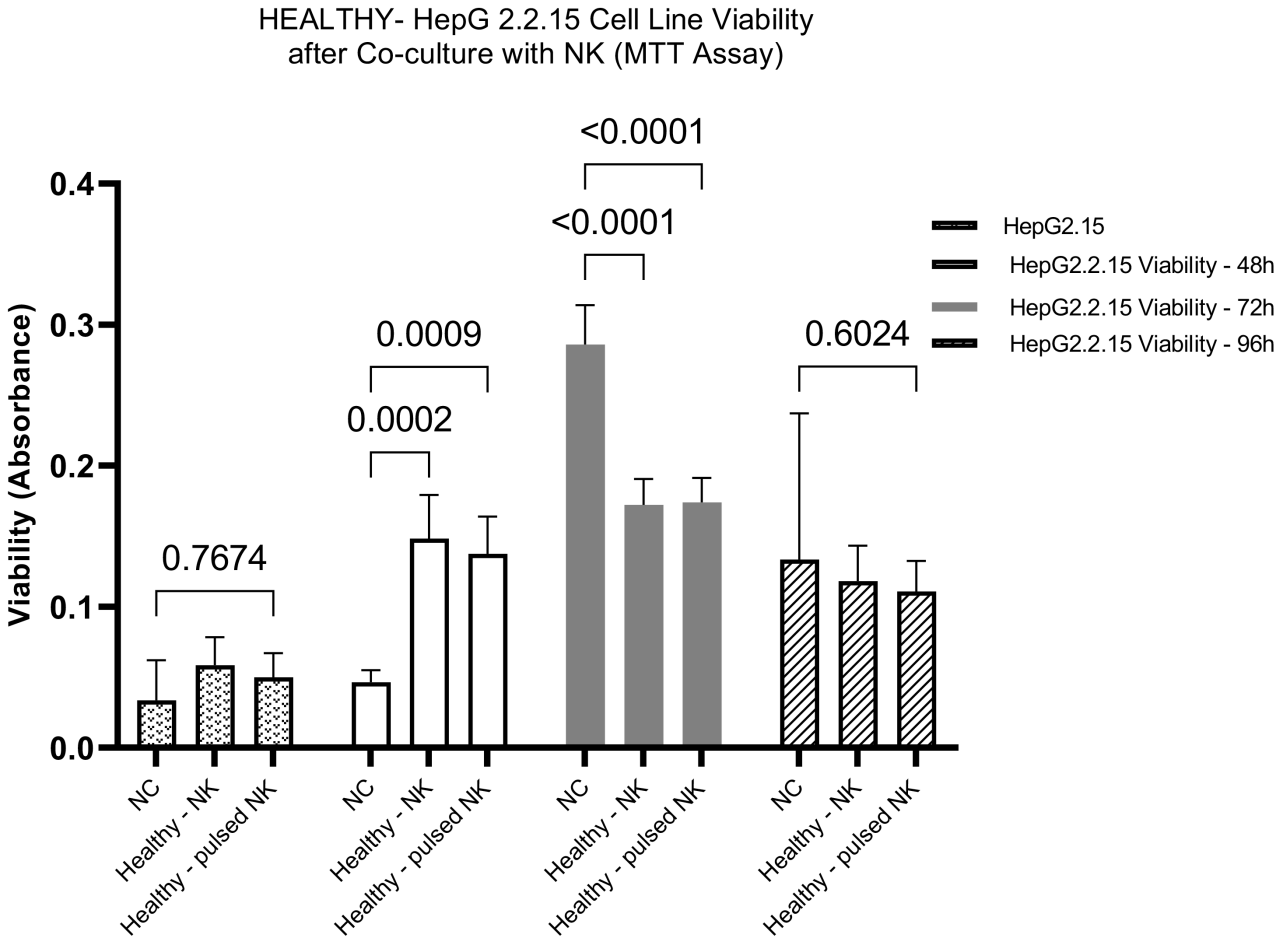
The bar graph illustrates the effect of HBVsvp-Stimulated NK cells on the viability of HepG2.2.15, highlighting changes over various time intervals. Data are presented as the mean ± SEM of three independent replicates. Statistical significance was assessed using two-way ANOVA to analyze the effects of time and treatment.

The cytotoxic potential of NK derived from healthy donors was assessed after stimulation with HBVsAg. The NK cells were co-cultured with HepG2.2.15 Cells as a model, and the HepG2.2.15 Cell viability was assessed at different time intervals; 24-Hour Co-Culture: Initially, the co-culture of HepG2.2.15 Cells with HBVsvp-stimulated NK cells showed a non-significant change in cell viability after 24 h. suggests This indicates that the immediate effect of the stimulated NK cells on HepG2.2.15 cells is minimal; 48-Hour Co-Culture A notable shift occurred at 48-h, where a significant increase in HepG2.2.15 cell viability was observed. suggesting an initial protective effect by activated NK cells.; 72-Hour Co-Culture Contrastingly, at 72 hours, a significant decrease in cell viability was reported. This long-term decrease may reflect the cytotoxic activity of NK cells becoming predominant, overcoming any initial supportive effects, and leading to the destruction of HepG2.2.15 cells; 96-Hour Co-Culture By 96 hours, the trend of decreasing cell viability continued, suggesting a sustained cytotoxic response from the NK cells over time. implying a sustained cytotoxic response from NK cells over time (**Figure 5**).

#### 3.3.3 Impact of HBVsvp-stimulated NK cells on HepG2.2.15 cells viability- CHB patients

**Figure 6:**
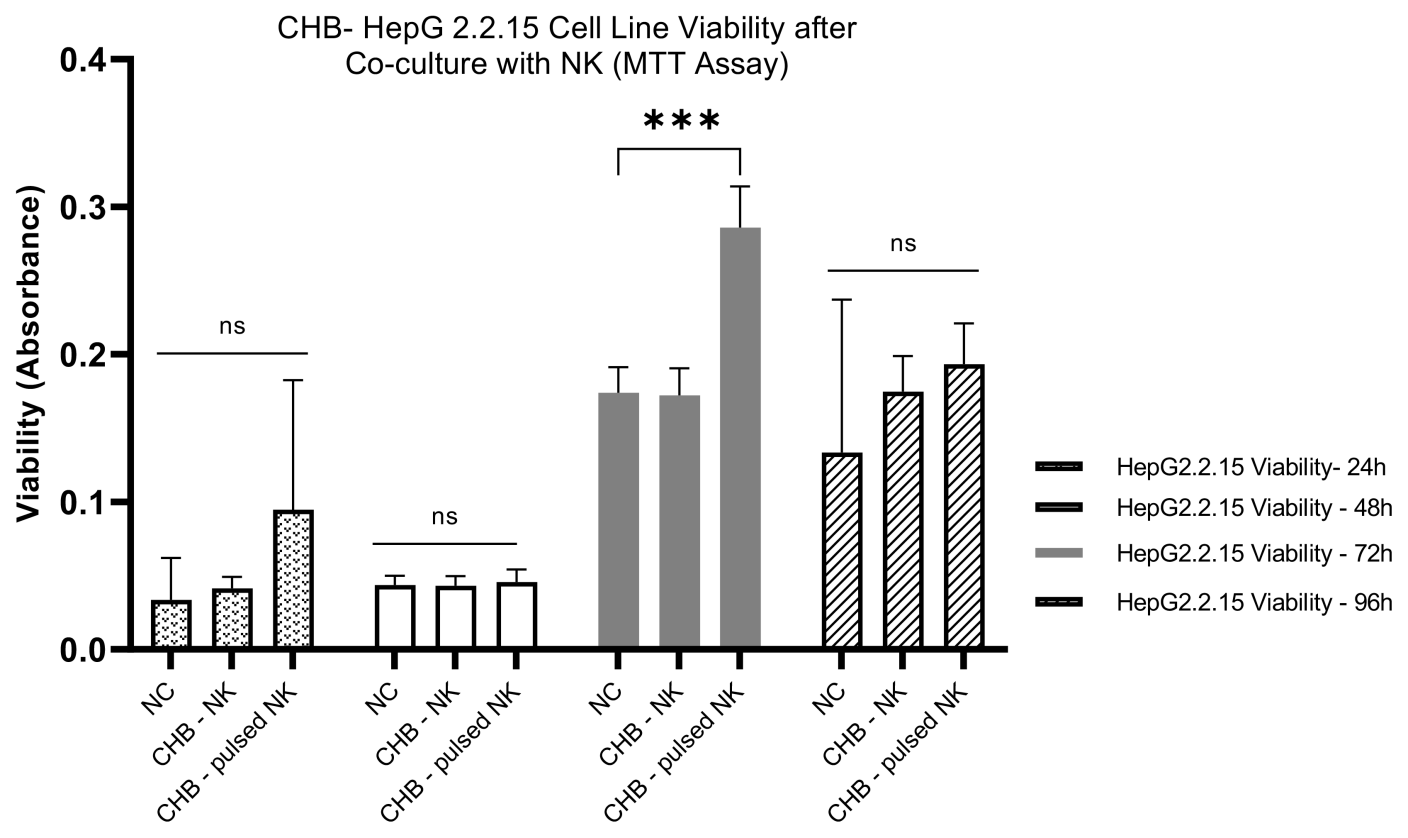
The bar graph explain the pattern effects of NK cells derived from CHB patients upon stimulation with HBVsvp on HepG2.2.15 cells cell viability over an extended period; The data are presented as the mean ± SEM of three independent replicates for each set of results. Two-way ANOVA tests were conducted to assess statistical significance, with p-values less than 0.001 (***). ns, non-significant.

The CHB-derived NK cells were co-cultured with HepG2.2.15 Cells as a model, and the HepG2.2.15 Cell viability was assessed at different time intervals *in vitro*. 24-Hour Co-Culture: After 24 hours of co-culturing with HBVsvp-stimulated NK cells, the HepG2.2.15 cells exhibited a non-significant change in viability. After 24 hours, there was a non-significant increase in HepG2.2.15 cells cell viability; 48-hour Co-Culture at 48 hours, there was not a significant shift in the viability of the cells. This suggests that the NK cells maintain a steady state with no marked impact on the HepG2.2.15 cells during this period; 72-Hour Co-Culture A significant increase in cell proliferation was noted at 72 hours P=0.0004, indicating a reduction in NK cell cytotoxic efficacy against HBV-infected cells upon stimulation with HBVsvp, could dampen NK cells are function that could affect their interaction with HepG2.2.15 cells., could be due to the chronic stimulation by HBVsvp; 96-Hour Co-Culture By 96 hours, there was a non-significant increase in cell viability. This suggests that while there is some level of NK cell activity, it is not sufficient to cause a significant change in the viability of the HepG2.2.15 cells (**Figure 6**).

#### 3.3.4 HepG2.2.15 cells viability mediated HBVsvp-stimulated NK cells viability- CHB vs healthy Donor

**Figure 7:**
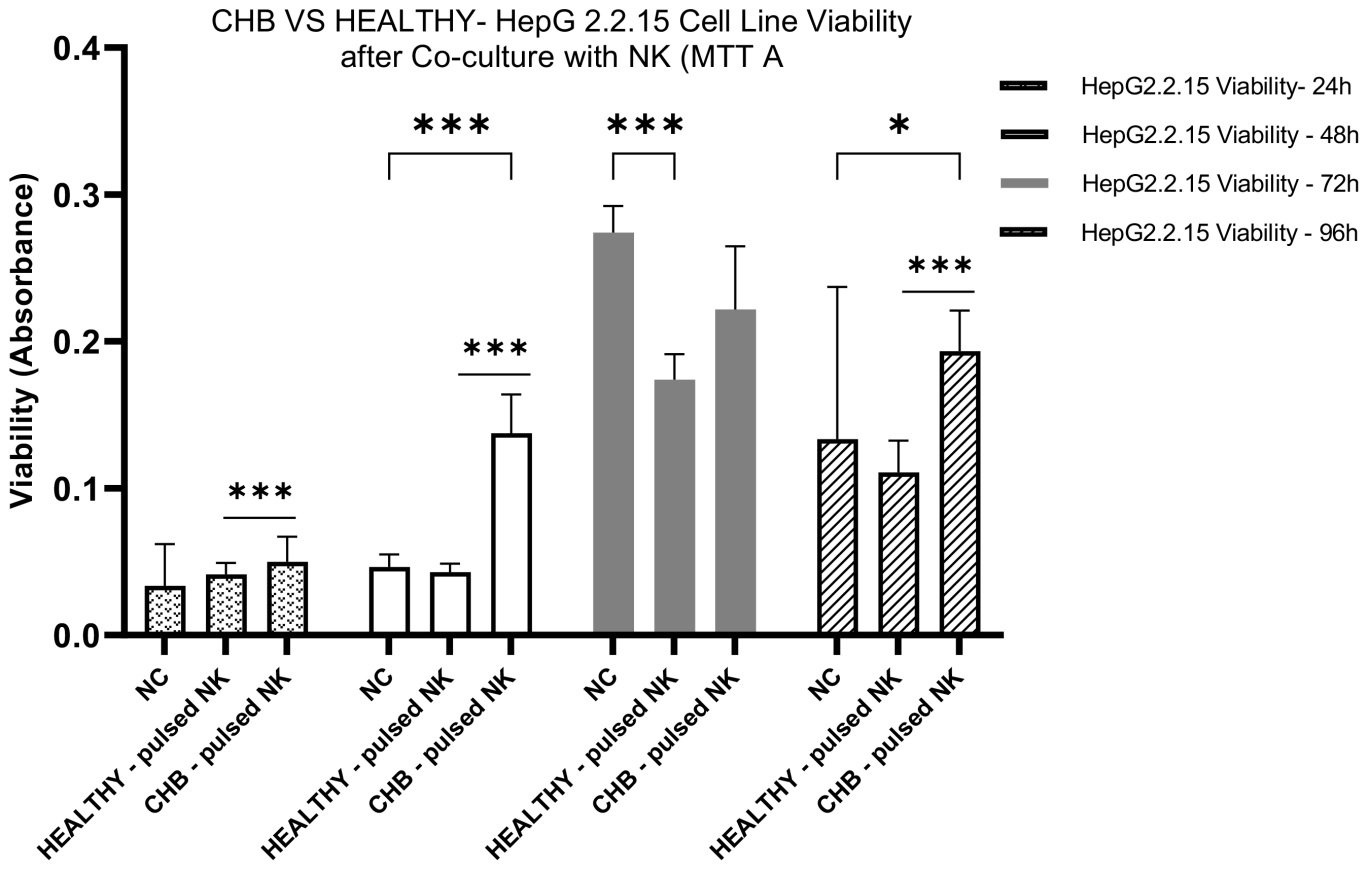
The bar graph illustrates the effects of HBVsvp stimulated NK cells derived from both healthy and CHB patients on the viability of HepG2.2.15 cells over time. Upon stimulation with HBVsvp, a distinct pattern of cell viability is observed. The data are presented as the mean ± SEM of three independent replicates for each set of results. Two-way ANOVA tests were conducted to assess statistical significance, with p-values less than 0.001 (***), and 0.05 (*).

Our comparative analysis assessed the impact of HBVsvp-stimulated NK cells on the viability of HepG2.2.15 cells. The analysis spanned both healthy individuals and CHB patients. We observed an increasing pattern in cell proliferation of HepG2.2.15 cells co-cultured with NK cells stimulated by HBVsvp from CHB patients at 24, 48, and 96 hours. This contrasted with the decreasing cell proliferation potentially leading to a cumulative effect that becomes more evident at later time points observed when co-cultured with stimulated NK cells from healthy donors. This suggests that NK cells from CHB patients, when stimulated by HBVsvp, may have diminished cytolytic capacity consequences by increasing HepG2.2.15 cell proliferation (**Figure 7**).

#### 3.3.5 HBVsvp quantification in co-cultured HepG2.2.15 Cells supernatant

**Figure 8:**
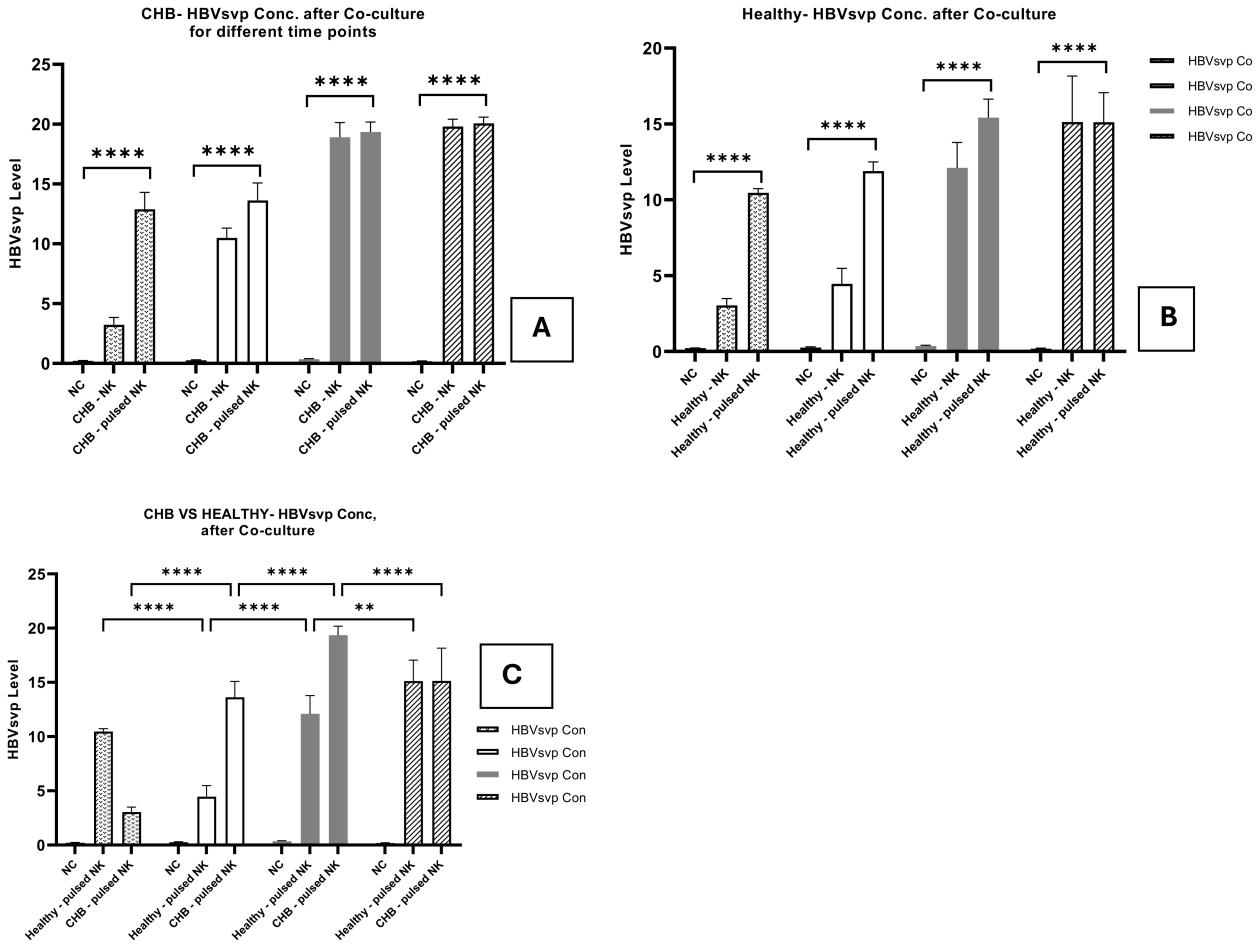
The bar graph Illustrates the comparative assessment of HBVsvp levels in the supernatant from HepG2.2.15 cocultured with NK cells stimulated by HBVsvp, highlights the temporal effects on HBVsvp production across various conditions: obtained from **(A)** healthy donors; **(B)**; CHB patients. **(C)** healthy donors VS CHB at 24, 48, 72, and 96 hours. The data are presented as the mean ± SEM of three independent replicates for each set of results. Two-way ANOVA tests were conducted to assess statistical significance, with p-values less than 0.0001 (****), and 0.01 (**) considered statistically significant.

We examined HBVsvp levels in the supernatant of a co-cultured system comprising HepG2.2.15 with HBVsvp-stimulated NK Cells derived from healthy donors at various period of time (24, 48, 72, and 96 hrs). The data unveiled a considerable increase (p<0.0001) in the levels of HBVsvp compared to a control group (**Figure 8A**). Furthermore, we observed elevated HBVsvp the supernatant’s level from HepG2.2.15 co-cultured with HBVsvp stimulated NK cells obtained from patients with CHB Moreover (p<0.0001), demonstrating the decreased cytotoxic suppressive effect of HBVsvp on NK cell activity through NK cell activation receptor downregulation and inhibitory receptor overexpression. (**Figure 8B**). Additionally, a comparative analysis of HBVsvp levels from stimulated NK cells across both healthy groups and CHB patients indicated a significant difference in HBVsvp levels produced from HepG2.2.15 for 48,72,96h. (**Figure 8C**).

#### 3.3.6 Altered TNF-α and production from HBVsvp stimulated NK-CHB evaluation

**Figure 9:**
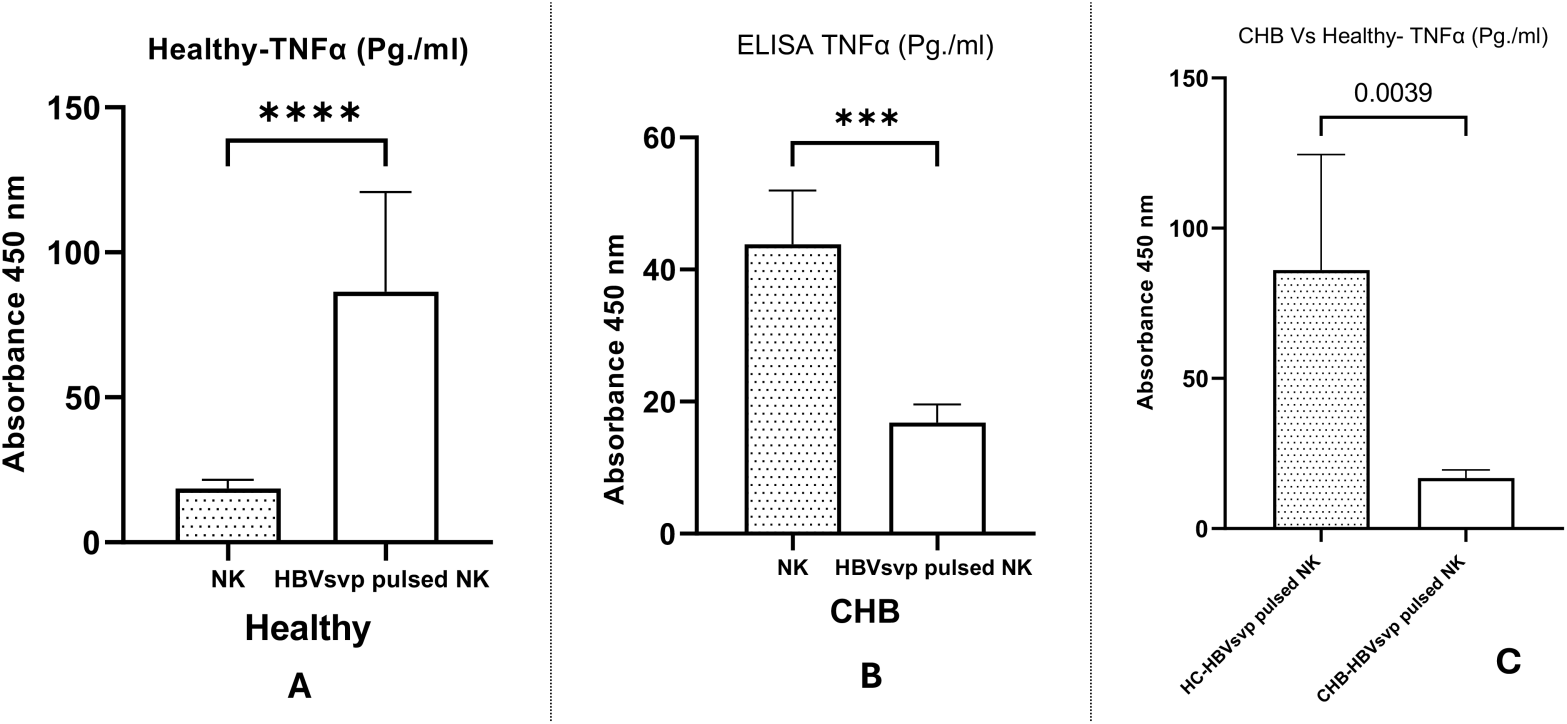
The bar graph displays the comparative levels of TNF-α secretion from NK cells of healthy donors and CHB patients post-HBVsvp pulsation. It elucidates the effects on TNF-α production across various conditions: **(A)** healthy donors; **(B)** CHB patients; and **(C)** a comparison between healthy donors and CHB patients. Data represents the mean ± SD from three independent experiments. The data are presented as the mean ± SEM of three independent replicates for each set of results. Unpaired two-tailed Student’s t-tests were conducted to assess statistical significance, with p-values less than 0.0001 (****), and 0.001 (***) considered statistically significant.

ELISA measurement of the TNF-α levels in the supernatants of NK cell cultures derived from healthy donors and CHB patients exposed to HBVsvp showed a significant elevation in TNF-α concentrations in the supernatants from NK derived from healthy donors after treatment HBVsvp, confirmed by (P < 0.0001) (**Figure 9A**), The presence of HBVsvp appears to stimulate the secretion of TNF- α effector cytokines, suggesting a more active role in early viral control. However, this exposure with HBVsvp on the other hand resulted in a significant decline in the functionality of NK cells, apparently from the significantly lower TNF-α levels released from NK in the CHB patient samples, (P < 0.001) (**Figure 9B**). Additionally, a comparative analysis of TNF-α levels from NK cells stimulated with HBVsvp across both healthy groups and CHB patients indicated a notable reduction in TNF-α, with a P value of P=0.0039 (**Figure 9C**), highlighting the difference in the inflammatory response to CHBV infection

#### 3.3.7 Modulation of IFN- γ secretion by HBVsvp - stimulated NK cells

**Figure 10:**
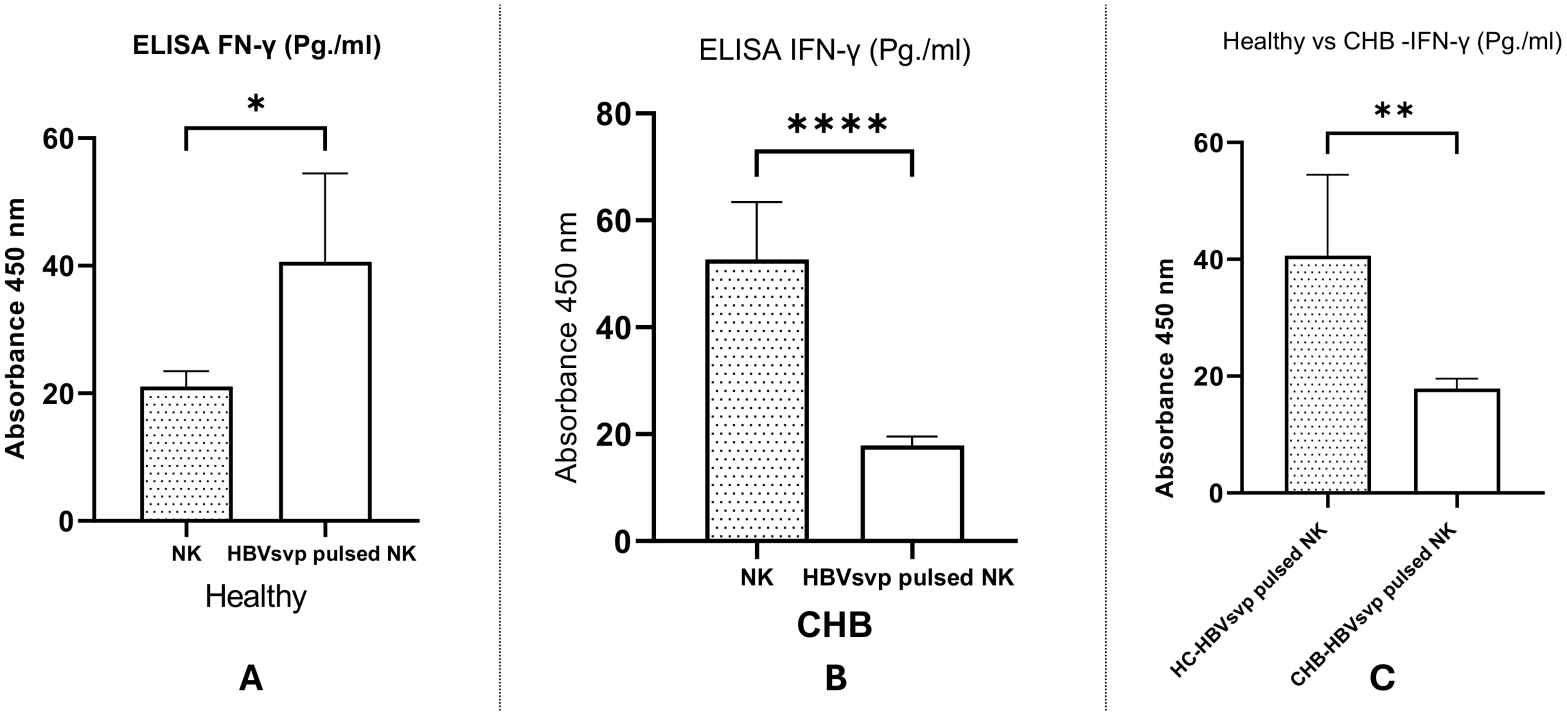
Shows a bar graph comparing the levels of IFN- γ secreted from NK cells from healthy donors and CHB patients After pulsation with HBVsvp, highlighting the effects of various NK cell conditions on IFN- γ production. across various conditions: **(A)** healthy donors; **(B)** CHB patients; **(C)** comparison between healthy donors and CHB patients. The data are presented as the mean ± SEM of three independent replicates for each set of results. Unpaired two-tailed Student’s t-tests were conducted to assess statistical significance, with p-values less than 0.0001 (****), 0.01 (**), and 0.05 (*) considered statistically significant.

A comparative analysis of IFN-γ secretion levels from NK cells derived from both healthy donors and CHB patients following stimulation with HBVsvp were examined. Our data revealed a significant increase in IFN- γ levels following treatment with NK cells with HBVsvp, as indicated by P <0.0001 (**Figure 10A**) suggesting that FN-γ a pivotal role in antiviral defense and immunoregulation in the initial stages of HBV infection. Conversely, HBVsvp treatment led to a notable reduction in NK cell function, evidenced by significantly lower IFN- γ levels in samples from CHB patients, with a P =0.0145, suggesting the defective production of IFNγ, particularly at the CHB stage (**Figure 10B**). Furthermore, comparing IFN- γ levels secreted from NK cells pulsed with HBVsvp from both donors and CHB patients, a significant decrease in IFN- γ was observed, with a P value of 0.0066 (**Figure 10C**), highlighting the differential impact of HBVsvp pulsation on NK cells’ ability to produce IFN-γ which associated with the altered activating-inhibitory receptor expression between chronic HBV and healthy donors

## 4 Discussion

The chronicity of HBV infection is a complex phenomenon that remains incompletely understood. However, accumulating evidence suggests that an inadequate immune response from the host has a crucial function in the establishment of chronic HBV carrier state (35). One potential mechanism underlying this impaired immunity is the immunosuppressive effects of HBV-coded antigens, which have been shown to interact with and suppress the function of key immune cells, such as monocytes, macrophages, and dendritic cells (36). NK cells, an additional essential part of the innate immune system, are known for their cytotoxic activity against virally infected cells and their ability to secrete immunomodulatory cytokines like IFN-γ (37). Clinical studies show a significant decline in NK cell function in chronic HBV patients compared to healthy controls (38). This suggests the fascinating prospect that HBV products could obstruct NK cell activity directly, contributing to the impaired immune response and the establishment of chronic HBV infection. Our study focused on investigating this possibility by examining the impact of HBsAg exposure on NK cell phenotype and effector functions *in vitro*. Our data suggests that HBsAg might directly interact with NK cells, leading to an immunosuppressive phenotype characterized by increased inhibitory receptors and decreased activating receptors, impaired cytotoxic activity, and diminished immunomodulatory IFN-γ - TNF-α production compared to healthy individuals. These findings collectively suggest that HBV antigens likely employ a multifaceted approach to suppress the host immune response, including observed NK cell dysfunction potentially contributing to the development of a chronic infection.

The considerable production of both complete and partial viral particles is a distinguishing feature of HBV replicating cells in the early stages of the viral infection. Complete mature virions, which are 42 nm spheres, consisting of glycoprotein-encased lipid membranes (L, M, and S), contain a nucleocapsid made up of viral polymerase, genomic DNA, and the hepatitis B core protein (39), usually be detected in infected patients’ blood at a rate of 10^9^/mL. On the other hand, incomplete HBVsvp comprises empty (genome-free) virions made of the viral capsid’s outer surface proteins without a genome. These are generally present in higher quantities than complete virions, approximately 100 times more (at a rate of 10^11^/mL). These SVPs mainly contain S proteins that efficiently induce secretion of the HBsAg (40), and presumably use HBsAg to act as a diminish the antiviral effect of neutralizing antibodies by using a decoy. This enhances infectious particles’ ability to access susceptible cells and promotes the immune tolerance necessary for sustaining long-term chronic infection (41). HBVsvp are immunogenic and share immunological determinants with full Dane particles, triggering an immune response (42). They contain HBsAg, which stimulates the production of protective anti-HBs antibodies (41) that are crucial for developing hepatitis B vaccines as they rely primarily on inducing immunity through HBsAg stimulation. consequently, these particles offer a model that acts as an immune stimulant to trigger the NK immune response, whether derived from CHB or healthy donors.

This study’s objective was to separate and describe HBVsvp by the invitro expression system (HepG2.2.15 cell line), as HBVsvp obtained from HepG2.2.15 cells displayed more pronounced distinctions than the historically and recently used HBVsvp obtained from infected individuals as it contains a range of host antibodies, either combined with the SVP or directly attached to it (43). Our molecular examination, utilizing a specific primer to identify the HBX gene presence, revealed that HBVsvp was an empty entity without the complete genome, as a 465 bp fragment of the HBX gene was not detected in our sample (**Figure 1**). This observation was compared with a prior study conducted in a similar context (43). After generating and characterizing the HBVsvp, we employed two approaches to separate NK cells. One involved employing flow cytometry for sorting, while the other relied on using Ficoll separation based on blood cell density. A comparison of both methods in examining marker expression on NK cells revealed no significant differences in purity or marker study outcomes. Consequently, we opted to proceed with the study without sorting due to financial constraints (34). Given that PBMCs are a common source of NK cells, It’s crucial to remember that the proportion of NK cells within PBMCs is typically low (around 5–20%) (44), which supports NK cell-derived PBMCs have an estimated yield of 22% and a purity level of around 64.97% according to our analysis (**Figures 3A, B**).

Immune cell exhaustion is a common feature in chronic infections and cancer, characterized by diminished effector functions, phenotypic alterations, reduced proliferation, impaired cytokine production, and decreased killing capacity (45). Studies have reported the immunosuppressive effects of HBV antigens, which manipulate DCs and Tregs. HBVsAg can suppress monocyte-derived dendritic cell differentiation (46), macrophages (47), impair the antigen-presenting ability of DCs, reducing their capacity to produce pro-inflammatory cytokines, leads to a more tolerogenic than immunogenic response which facilitates viral persistence (48). Furthermore, HBsAg can impair the ability of DCs to induce NK cell proliferation and activity (49). Additionally, HBV can induce DCs to secrete immunosuppressive cytokines like IL-10 and TGF-β (50) contributing to Tregs expansion and effector T cell inhibition, as well as large amounts of HBsAg might induce T cell anergy, leading to decreased antibody-mediated neutralization of HBV and generalized hyporesponsiveness toward pathogens (47).

In the context of NK cells exhaustion by HBV antigen (HbsAg), has been shown to have multifaceted effects on the phenotype of NK cells. These effects can vary depending on the stage of infection (acute or chronic), antigen level, and individual immune response (51). Consistent with previous studies (28, 52–54), but in opposite to others (22, 55, 56), we revealed that NK cells phenotypic profiling in the case of NK cells from CHB patients, we observed significantly downregulated activating receptors NKG2D and NKp46 (P Value = 0.022); on the other hand, the CD94 inhibitory marker, significantly (p = 0.029) upregulated after HBVsvp pulsation) *in vitro* harvested from HepG 2.2.15(when compared to downregulated inhibitory marker and upregulated activating marker on NK cells derived from healthy donors (**Figures 4A–C**). This shift in the expression imbalance involving both inhibitory and activating receptors on NK cells in CHB can be indicative of cell exhaustion (57), and correlates with recurrent prolonged exposure to HBVsvp antigen, a phenomenon that is like what has been observed in T cells during chronic viral infections (12). The modifications consequences in the phenotype of NK cells could significantly affect their ability to combat viral infections or malignancies by reducing their cytotoxic activity (58), impairing antitumor response (59), and decreasing their capacity to release IFN-γ (60), contributing to the overall immune suppression, and may also play a role in the progression of diseases such as HBV infection. However, it is important to note that while our invitro study demonstrates a potential relationship between HBsAg levels and the impairment of NK receptor expression, further *in vivo* studies or clinical data would be necessary to confirm this correlation in clinical setting. The potential mechanisms related to HBV antigens might implicate the inhibition of the NF-κB and p38 MAPK pathways, which are known to be significant for NK cell activation and the generation of cytokines, has been demonstrated (61). Additionally, the phosphorylation of STAT1, a regulator of NK cell activation and the generation of cytokines, was found to be inhibited by HBsAg and HBeAg in NK-92 cells (62). Prior research suggested that contact-dependent processes, such as the binding of the HCV envelope protein to CD81 on the surface of NK cells, may prevent the functional activation of NK cells (63). contributing to the development of persistent infection and the failure of viral clearance. Therfore, further clarification required to explore the downstream intracellular cascade impacted by altered NK receptor expression. This underscores the importance of developing therapeutic approaches that can adjust these NK receptor alterations and its downstreaming cascade to enhance NK cell-mediated immune responses.

As a non-cytopathic virus, HBV can inflict different levels of tissue damage by inducing a protective immune response that can be harmful as well as protective. The immune system destroys virus-infected cells to eradicate intracellular viruses. As a result, the destruction of infected cells by the immune system can either effectively terminate infection or lead to persistent disease (64). For this study, the HepG2.2.15 cell line (transfected with HBV surface region) for SVPs production was chosen as an *in vitro* model system to investigate its interaction with NK cells concerning HBV infection and NK effector capacities analyses (cytotoxicity and production of IFN-γ, TNF-α cytokines). In our study, the cytotoxic potential of HBVsvp stimulated NK cells derived from healthy donors was evaluated, The NK cells were co-cultured with HepG2.2.15 cells as a model, and the viability of HepG2.2.15 cells was assessed at different time intervals. Initially, there was a non-significant change in HepG2.2.15 cell viability after 24 hours of co-culture, indicating a minimal immediate effect of stimulated NK cells on HepG2.2.15 cells. A significant increase in HepG2.2.15 cell viability was observed at 48 hours, suggesting an initial protective effect by activated NK cells. In contrast, at 72 hours, a significant decrease in cell viability was reported, indicating that the cytotoxic activity of NK cells became predominant, leading to the destruction of HepG2.2.15 cells. By 96 hours, the decreasing trend in cell viability continued, suggesting a sustained cytotoxic response from NK cells over time (**Figure 5**). These results demonstrate a dynamic interplay between HBVsvp-stimulated NK cells and HepG2.2.15 cells, with an initial protective effect transitioning to a cytotoxic response over time. On the other hand, we tried to find the impact of HBVsvp-stimulated NK cells from CHB patients on HepG2.2.15 cell viability through *in vitro* co-culturing. After 24 hours of co-culturing with HBVsvp-stimulated NK cells, there was a non-significant change in HepG2.2.15 cell viability. A non-significant increase in cell viability was observed, indicating a subtle effect on cell viability. At 48 hours, no significant change in cell viability was observed, suggesting that NK cells maintained a steady state with no marked impact on HepG2.2.15 cells during this period. A significant increase in cell proliferation was noted at 72 hours (P=0.0004), indicating a decrease in the cytotoxic activity of NK cells against HBV-infected cells upon stimulation with HBVsvp. This decrease in cytotoxic activity could potentially affect the interaction of NK cells with HepG2.2.15 cells and may be attributed to chronic stimulation by HBVsvp. By 96 hours, there was a non-significant increase in cell viability, suggesting that while there is some level of NK cell activity, it may not be sufficient to cause a significant change in the viability of HepG2.2.15 cells (**Figure 6**). These results highlight the complex interplay between HBVsvp-stimulated NK cells from CHB patients and HepG2.2.15 cell viability over time, indicating varying effects at different time points. By Comparing the impact of HBVsvp-stimulated NK cells on the viability of HepG2.2.15 cells between CHB patients and healthy donors, the following results were observed: An increasing pattern in cell proliferation of HepG2.2.15 cells was noted when co-cultured with NK cells stimulated by HBVsvp from CHB patients at 24, 48, and 96 hours. In contrast, a decreasing trend in cell proliferation was observed when co-cultured with stimulated NK cells from healthy donors (**Figure 7**). This trend potentially leads to a cumulative effect that becomes more evident at later time points. The results shed light on the differential impact of HBVsvp-stimulated NK cells from CHB patients and healthy donors on the viability of HepG2.2.15 cells. This interplay could be explained by activating and inhibitory receptor expression alteration, resulting in a diminished cytolytic capacity, evidenced by increased HepG2.2.15 cell proliferation, or by activating receptor-ligand interactions modulation, or by the altered expression of NK cell ligands on infected hepatocytes when exposed to HBVsAg, potentially enabling these cells to evade surveillance by NK cells (65). emphasizing the complex interplay between immune responses and cell viability in the context of CHB infection.

The HBsAg is a key marker for monitoring the progression of HBV infection. According to the research, the presence or absence of HBsAg, along with the HBV DNA level and ALT level, helps diagnose the phase of chronic HBV infection (66). In our study, a comparative analysis of the HBVsvp levels in the supernatant of a co-culture system involving HepG2.2.15 cells with HBVsvp-stimulated NK cells from healthy donors and CHB patients indicated a significant difference in HBVsvp levels produced from HepG2.2.15 for 48, 72, and 96 hours (increase (p<0.0001) in HBVsvp levels in CHB) (**Figure 8C**), attributed to the downregulation of activating receptors and upregulation of inhibitory receptors on NK cells. This demonstrated that HBsAg level can be used to predict the nature of NK diminished cytotoxicity against HBV-infected cells. Furthermore, measurement of HBsAg may aid in improving future therapy methods for both immune-modulator therapy and nucleos(t)ide analogues (67).

IFN- γ and TNF-α play a critical role in the management of hepatitis B virus infections. They are involved in the recruitment and activation of immune cells such as T cells, NK cells, and macrophages, which in turn produce cytokines with antiviral properties. These cytokines stimulate T cell development to acquire antiviral functions, increase MHC expression on liver cells for improved antigen presentation, and directly execute antiviral actions for effective infection control (68). In our study, a comparative analysis of TNF-α levels from HBVsvp stimulated NK cells across both healthy groups and CHB patients indicated a notable reduction in TNF-α in the CHB group, with a P value of P=0.0039 (**Figure 9C**), which is consistent with NK cells activation during acute HBV infection before the onset of adaptive immunity (69). Furthermore, comparing IFN- γ levels secreted from NK cells pulsed with HBVsvp from both donors and CHB patients, a significant decrease in IFN- γ was also observed with the CHB group, with a P value of 0.0066 (**Figure 10C**), highlighting the differential impact of HBVsvp pulsation on NK cells’ ability to produce IFN-γ which associated with the altered activating-inhibitory receptor expression between chronic HBV and healthy donors which in the same line with a decreased capacity to produce cytokines such as IFN-γ and TNF-α in the chronic phase of the disease (70). Understanding how altered cytokine production by NK cells affects the immune response during CHB infection can provide insights into potential NK-based immunotherapies.

During primary HBV infection, NK cells play a crucial role in the initial control of the virus through their cytotoxic capabilities and the production of effector cytokines (71). This early NK cell response facilitates the transition to adaptive immunity, where CD8+ T cells, assisted by NK cells, become key for clearing HBV from the liver (72).

As chronic hepatitis B progresses, NK cells exhibit fluctuating proportions, dysregulated pgynotypic receptor expression, increased inhibitory molecules, sustained/heightened cytotoxicity linked to liver damage, and reduced antiviral cytokine production (45). Our findings suggest that the effects of HBVsvp stimulation on NK cells derived from CHB patients are in line with these reported results. Furthermore, the impairment of NK cell function in CHB can also impact the function of CD8+ T cells, the primary effectors responsible for viral clearance during acute HBV infection (73). In chronic HBV, CD8+ T cells exhibit an exhausted phenotype, being reduced in number and characterized by sustained expression of inhibitory receptors, diminished cytotoxicity, and impaired cytokine production (74). Furthermore, the diminished CD8+ T cell population in CHB patients is partially attributed to the downregulation of activating receptors on NK cells. This leads to reduced NK cell responsiveness and decreased production of antiviral cytokines like IFN-γ, which are crucial for stimulating and supporting T cell responses (75), either directly or indirectly by enhancing DCs maturation and IL-12 production. The resulting less supportive environment due to the reduction in NK cell activation and cytokine production contributes to the diminished CD8+ T cell population in these patients (23). NK cells in CHB can directly suppress HBV-specific CD8+ T cells via TRAIL-mediated apoptosis and contribute to T cell exhaustion through the production of immunosuppressive cytokines like IL-10 and TGF-β (76). This regulatory function of NK cells, while potentially important for controlling immune-mediated damage, can become detrimental in chronic infections, hindering the ability of T cells to effectively clear the virus.

B cells play multifaceted roles beyond antibody production, including antigen presentation and immune regulation that impact HBV persistence and immune toleran`ce (74). NK cells bidirectionally modulate B cell responses during viral infections through various mechanisms. Activated NK cells secrete IFN-γ, which can enhance B cell activation, antibody production, and IgG class switching (77). IFN-γ also indirectly supports B cell responses by recruiting DCs crucial for antigen presentation and B cell activation (78). Studies suggest that NK cells might directly engage with B cells and other cytokines like TNF-α and cell surface receptors, potentially influencing their activation and antibody production (79). Our findings indicate that chronic HBV patients’ NK cells exhibited significantly impaired function when exposed to HBVsvp, a phenomenon termed “exhaustion.” This dysfunction, characterized by reduced IFN-γ and TNF-α production and altered receptor expression, likely impairs NK cells’ ability to effectively stimulate B cells and promote antibody responses. This further exacerbates the pre-existing dysfunction of HBsAg-specific B cells, creating a cycle that facilitates viral persistence.

Chronic exposure to HBsAg can indirectly modulate NK-B cell interactions. HBsAg presentation by B cells to CD8+ T cells leads to destruction of HBsAg-specific B cells, hindering their ability to produce protective anti-HBs antibodies and potentially disrupting further NK-B cell interactions (74). In CHB patients, HBsAg-specific B cells are dysfunctional, exhibiting atypical memory B cell characteristics with high inhibitory receptor PD-1 expression. This impairs their ability to proliferate, differentiate into antibody-secreting cells, and generate protective anti-HBs antibodies, leading to low antibody levels (80). Anti-HBs antibodies are crucial for neutralizing viral particles, blocking hepatocyte entry, and directing antibody-dependent cellular cytotoxicity (ADCC) to eliminate infected cells (81). Collectively, the results of our investigation demonstrate that HBsAg-mediated dysregulation of NK cell function precipitates a cascade of events that culminates in a compromised and ineffective adaptive immune response, involving both B and T lymphocytes, against CHB virus infection.

## 5 Limitation

A potential limitation of this study is the relatively small sample size (n=10). While our findings demonstrate significant differences between CHB patients and healthy controls, a larger sample size could provide increased statistical power and enhance the generalizability of our results. Future studies with a larger cohort are warranted to further validate these findings, explore the potential clinical implications, establish utility as reliable biomarkers, and underlying mechanisms of HBVsvp stimulated NK cell exhaustion in greater detail. Moreover, our study examined cytotoxicity assay and did not examine NK cell degranulation capacity or markers of functional exhaustion in NK cells. To gain a comprehensive understanding of NK cell responses in chronic HBV infection, future studies should incorporate the analysis of NK cell degranulation or markers of functional exhaustion as well, as this would provide additional insights into the influence of SVPs on the ability of NK cells to release cytotoxic granules, which is a crucial mechanism for target cell lysis. In the current study, we also have provided valuable insights into the impact of HBVsvp exposure on NK cell function and receptor expression. However, we acknowledge that while we selected patients in the chronic phase, the duration of HBV infection was not systematically assessed. Further retrospective and subgroup (with short-term (<5 years) and long-term (≥5 years) HBV infection) analyses are needed to investigate the relationship between HBV infection duration and NK cell responses required, since this could provide valuable insights into optimizing therapeutic approches for chronic HBV patients.

## 6 Conclusion

In summary, our work shed light on the ability of HBV antigen to affect NK cell function, namely its ability to inhibit NK cell activation, cytokine generation, and cytotoxic reduction. This implies that HBV evades immune surveillance by the direct immuno-suppressive impact of circulating HBsAg particles (represented by *in vitro* HBVsvp) on NK cell activity, potentially influencing treatment results, illness persistence, and severity. Additionally, gaining a better understanding of the underlying processes that lead to NK cell exhausion, should enhance our comprehension of the fundamental biology of NK cells during the chronic HBV infection. Modulating NK cell receptor expression, including both activating and inhibitory receptors through NK cell-based immunotherapy, may represent a promising future approach to restore the functionality of dysfunctional NK cells and enhance their proliferation and effector functions. This could be a valuable strategy to combat HBV infection and other chronic infections. Further research in clinical settings are needed to fully understand the therapeutic potential and optimize the safety profiles. Furthermore, although the primary focus of our investigation was on the impact of HBsAg on NK cell performance, it would be beneficial to conduct comparative studies in the future to examine the effects of other viral proteins, such as HbeAg (alone or in conjuction with HBsAg), on NK cell function. These studies could uncover unique or overlapping pathways of immune regulation. Incorporating an experimental approach involving co-culturing HBVsvp-sensitized NK cells with CD8+ T cells represents an important area for future investigation, as understanding the mechanisms by which HBVsvp modulate the functions of both NK cells and CD8+ T cells could elucidate novel therapeutic targets for enhancing HBV antiviral immunity. While our study was designed to focus primarily on the immediate effects of HBV subviral particles on NK cell phenotype and function, Expanding the analysis to include different stages of chronic hepatitis B, such as immune tolerant, immune reactive, symptomatic, and inactive carriers, would provide crucial insights into how HBV subviral particles interact with NK cell immune responses over the course of the disease. This comprehensive understanding is essential for managing disease progression and guiding effective treatment strategies.

## Data Availability

The original contributions presented in the study are included in the article/supplementary material. Further inquiries can be directed to the corresponding authors.
